# Stimulating Neuroplasticity Through Language Learning: Innovative Pathways to Healthy Aging in Older Adults

**DOI:** 10.1093/geroni/igaf122.2911

**Published:** 2025-12-31

**Authors:** Ladan Ghazi Saidi, Yunju Im, Yijun Lu, Cary Savage, Douglas Schultz

**Affiliations:** University of Nebraska Kearney, Kearney, Nebraska, United States; University of Nebraska Medical Center, Omaha, Nebraska, United States; University of Nebraska Medical Center, Omaha, Nebraska, United States; University of Nebraska-Lincoln, Lincoln, Nebraska, United States; University of Nebraska-Lincoln, Lincoln, Nebraska, United States

## Abstract

Healthy aging relies on innovative interventions to sustain cognitive abilities. Language learning is a novel approach to stimulate cognitive function and neuroplasticity. We examine structural brain changes, focusing on cortical thickness variations, in older adults participating in new language acquisition. Healthy monolingual participants (n = 41) aged 60 to 80 residing in a monolingual environment engaged in an online language-learning program. The intervention involved 90-minute daily sessions, five days per week, over four months. Brain cortical thickness was measured before and after the intervention and compared. Behavioral results suggest that all participants successfully learn their language of choice with an average performance score of 96% in the post intervention proficiency test. Neuroimaging analyses reveal a weak trend (p < 0.01, uncorrected) indicating structural brain alterations associated with language learning. Changes were observed in the left middle frontal sulcus, the right superior frontal sulcus, and the right transverse temporal sulcus—regions implicated in working memory, attention, cognitive control, executive functions, and language processing. Although these findings did not survive multiple comparisons correction (FWE correction), they suggest structural changes in brain areas crucial for cognitive performance and healthy aging, particularly in executive functions. These results indicate that four months of language learning can induce structural neuroplasticity in older adults, with particularly notable changes in the right hemisphere. While these findings support the potential of language acquisition as an intervention for enhancing brain plasticity in aging, greater effects may be achieved with a higher dosage or longer duration of training, warranting further investigation.

